# Naturally Occurring Alpha-Synuclein Autoantibodies in Parkinson’s Disease: Sources of (Error) Variance in Biomarker Assays

**DOI:** 10.1371/journal.pone.0114566

**Published:** 2014-12-03

**Authors:** Sebastian Heinzel, Maike Gold, Christian Deuschle, Felix Bernhard, Walter Maetzler, Daniela Berg, Richard Dodel

**Affiliations:** 1 Department of Neurodegeneration, Hertie Institute for Clinical Brain Research (HIH), University of Tuebingen, Tuebingen, Germany; 2 Department of Neurology, Philipps-University Marburg, Marburg, Germany; 3 German Center for Neurodegenerative Diseases (DZNE), Tuebingen, Germany; University of Nebraska Medical center, United States of America

## Abstract

Alpha-synuclein (α-Syn) plays a pivotal role in the pathophysiology of Parkinson’s disease (PD), which can partly be modulated by innate and adaptive immune functions, and vice versa. Here, naturally occurring α-Syn autoantibodies (α-Syn-nAbs) may be effective against α-Syn pathoetiology and may serve as a PD biomarker. However, serum and cerebrospinal fluid α-Syn-nAbs levels still lack consistent evidence as required for a reliable PD biomarker. Serum and cerebrospinal fluid α-Syn-nAbs levels of 66 PD patients and 69 healthy controls were assessed using a validated ELISA assay. Moreover, potential sources of error variance including unspecific ELISA background signals, free serum hemoglobin concentrations, α-Syn plate coating procedures, and differences in α-Syn-nAbs standards, were investigated. PD patients and controls did not differ in serum (p = .49) nor cerebrospinal fluid (p = .29) α-Syn-nAbs levels. Interestingly, free serum hemoglobin concentrations were negatively correlated with α-Syn-nAbs levels in controls (Spearman 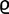 = −.41, p<.001), but not in PD patients (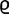 = .16, p = .21). ELISA α-Syn plate coating procedures impacted inter-assay variability (same day coating: 8–16%; coating on different days: 16–58%). α-Syn-nAbs standards from different purification batches differed regarding optical density measured in ELISAs suggesting differences in α-Syn affinity. While α-Syn-nAbs levels may represent a potential PD biomarker, several methodological issues have to be considered to increase reproducibility of α-Syn-nAbs findings. Further studies using standardized protocols minimizing sources of error variance may be necessary to establish a reliable PD α-Syn-nAbs biomarker.

## Introduction

Alpha-synuclein (α-Syn) plays a pivotal role as the conversion of its soluble form into insoluble α-Syn aggregates represents a key event in the pathogenesis of Parkinson’s disease (PD) [1], [2]. α-Syn aggregates into neurons and may lead to Lewy body pathology and the progressive degeneration of dopaminergic neurons [3], [4]. Reduced total α-Syn [5]–[8], and increased α-Syn oligomer levels [9], in cerebrospinal fluid (CSF) of PD patients compared to healthy controls (HC) have repeatedly been shown, which may allow differentiation on the basis of this biomarker. However, inconsistent evidence of blood serum or plasma α-Syn level differences between PD patients and HC have been reported with non-significant differences [10]–[14], and increased [15]–[17] and decreased [18] α-Syn levels in PD patients compared to HC.

Alpha-synucleinopathy has been linked to dysfunctional processes in both, adaptive and innate immunity [19], [20], and therapeutic immunization strategies may represent one avenue to modulate PD pathogenesis [21]. Active immunization with α-Syn or direct transfer of antibodies against α-Syn has been shown to be effective against Lewy body pathology in mice [22], [23]. Passive immunization strategies involving naturally occurring α-Syn autoantibodies (α-Syn-nAbs) have been suggested for the treatment of PD patients [24]. Principally, as shown for nAbs binding to β-amyloid and prion aggregates, respectively, nAbs are crucial for microglial phagocytosis of antibody-antigen complexes while also inhibiting pathological protein aggregation [25]–[28]. Therefore, nAbs may represent an important physiological mechanism against toxic protein aggregates, which are common in a variety of neurodegenerative diseases.

α-Syn-nAbs have been repeatedly investigated as a biomarker in PD, however, as for α-Syn levels evidence showing differences in α-Syn-nAbs in serum or plasma between PD patients and HC have been largely inconsistent. While some studies showed non-significant differences between these diagnostic groups [14], [29]–[31], also increased [32]–[34] and decreased [35] α-Syn-nAbs levels in PD patients compared to HC have been reported. For patients with Alzheimer’s disease or Lewy-body dementia, another alpha-synucleinopathy, increased serum α-Syn-nAbs compared to HC have been shown [36].

The disparity among these studies may have resulted from several factors, which introduce error variance in biomarker data or limit the comparability of studies. For instance, (1) sample characteristics including PD subtype and diagnosis, disease severity, age and sample size, (2) biological sample quality, e.g. contamination with erythrocytic α-Syn [13], [37], [38], and (3) differences in α-Syn-nAbs enzyme-linked immunosorbent assay (ELISA) protocols.

To address some of these issues impacting α-Syn-nAbs ELISA assays and data interpretability, the present study assessed serum and CSF α-Syn-nAbs levels in PD patients and HC as well as the impact of potential sources of error variance affecting α-Syn-nAbs data. We hypothesized to show decreased serum (and CSF) α-Syn-nAbs levels in PD patients compared to HC (consistent with a previous study using largely the same ELISA protocol; [35]). Moreover, investigated methodological aspects possibly increasing α-Syn-nAbs error variance and thereby contributing to inconsistencies of PD α-Syn-nAbs findings were (1) unspecific ELISA background signals, (2) free serum hemoglobin concentrations, (3) α-Syn plate coating procedures, and (4) α-Syn affinity differences in α-Syn-nAbs standards.

## Methods

### Patients and samples

The study was approved by the ethical committee of the Medical Faculty of the University of Tuebingen (389/2013BO2). All procedures were in accordance with the Declaration of Helsinki in its latest version, and all subjects gave written informed consent. PD patients (n = 66) were recruited from the ward and the outpatient clinic of the Department of Neurodegeneration of the University of Tübingen, Germany. PD patients were diagnosed by specialists in the field of neurodegenerative movement disorders based on the UKPDS diagnostic criteria [39] and disease stage was indicated according to Hoehn & Yahr (H&Y) [40]. 26 of the PD patients were categorized as having idiopathic Parkinson’s syndrome (IPS) without further classification, 15 IPS of the equivalent type, 19 IPS of the hypokinetic rigid type, and 6 IPS of the tremor-dominant type. Healthy controls (HC; n = 69) had no history or signs of any neurologic disorder. PD patients and HC did not differ in age (t_133_ = −.54, p = .59) nor gender (χ^2^ = .16, p = .70; HC: 28 females/41 males, PD: 29/37). While for serum ELISAs and free hemoglobin (Hb) analyses samples of all 66 PD patients and 69 HC were performed, CSF samples were only available for 59 PD patients and 46 HC.

Serum and CSF samples were collected in polypropylene tubes (1.5 mL for Serum: neoLab, Heidelberg, Germany; 0.5 mL for CSF: Sarstedt, Nümbrecht, Germany) and after centrifugation (serum: 2000x g, CSF: 4000x g; 4°C, 10 min.) samples were stored at −80°C within 60 min. Sample storage time was comparable between the investigated groups for both serum (mean ± standard deviation (SD): PD: 3.5±2.0 years, HC: 3.1±1.9; p>.1) and CSF samples (PD: 3.4±1.9, HC: 2.9±1.9; p>.1). Photometric Hb concentration measurements at 545 nm were conducted using ADVIA 1800 (Siemens Healthcare, Erlangen, Germany) yielding a sensitivity of c(Hb) ≥0.01 g/L.

### α-Syn-nAbs standards

The standards for ELISA tests consisted of α-Syn-nAbs isolated from two different preparations of one batch of human intravenous immunoglobulin G (IVIg) (Octagam10%; a gift from Octapharma, Lachen, Switzerland). α-Syn-nAbs extraction for standard #1 and #2 was performed using affinity chromatography as previously described [35]. Briefly, a column was packed with 2 mL NH_2_-activated resin (PIERCE Biotechnology, Rockford, IL, USA), labeled with 1 mg recombinant α-Syn (rPeptide, Bogart, GA, USA), equilibrated, and washed with phosphate-buffered saline (PBS, pH 7.4). After passing purified IVIg through the column, 16 fractions were eluted with glycine buffer (pH 2.8). The main fractions containing the highest amount of α-Syn-nAbs were pooled and their concentration determined using the NanoDrop spectrometer (Nanodrop1000, PeqLab, Erlangen, Germany). Pooled α-Syn-nAbs were stored until use at −20°C. Preparation of standards involved were prepared by diluting affinity-purified α-Syn-nAbs from IVIg in dilution buffer (5% BSA with 0.1% Tween-20). The specific binding of α-Syn-nAbs to recombinant α-Syn was detected using dot blots. Briefly, 5 µl of PBS diluted recombinant α-Syn (200, 100, 50, 25, 12.5, and 6.25 µg/mL) was applied to nitrocellulose membrane stripes (pore-size 0.45 µm; Millipore, Bedford, Mass., USA), after 30 min drying, non-specific sites were blocked using Roti-Block blocking reagent (Roth, Karlsruhe, Germany) for one hour at room temperature (RT). The membrane was incubated with purified α-Syn-nAbs (2 µg/mL diluted in blocking reagent) over night. After washing the membranes three times for 5 min using PBS-0,05%Tween, the detection antibody anti Human-HRP (1∶500.000 dilution in RotiBlock (25 ml); Pierce, Rockford, IL, USA) was added and after 1 hour at RT membranes were washed again three times for 5 min. After adding The chemiluminescent substrate (SuperSignal West Dura, Pierce, Rockford, IL, USA) was added to the membrane and after 3 min at RT exposed to an X-ray film in the dark room for 10 min.

### α-Syn-nAbs ELISA

High-bind 96-well ELISA 2HB Immunolon plates (Thermo Scientific, Rochester, NY, USA) were used. Half of the wells were coated overnight with 50 µL/well recombinant α-Syn (3 µg/mL) (rPeptide, Bogart, GA, USA) in phosphate buffered saline (PBS, pH 7.4; Dulbecco, PAA Laboratories, Linz, Austria) at 4°C, whereas uncoated wells only contained PBS to determine the unspecific background signal. Wells were blocked using Roti-Block blocking reagent (Roth, Karlsruhe, Germany) with 0.1% Tween-20 (Applichem GmbH, Darmstadt, Germany) for 2 hours at RT. Serum samples were diluted 1∶50 and CSF samples 1∶4. Plates with α-Syn coated and uncoated wells were washed 3 times with 300 µL washing buffer (PBS with 0.05% Tween-20) using an Amersham Biotrak II Plate Washer (GE Healthcare Europe, Freiburg, Germany). Plates were incubated with triplicates (50 µL/well) of standards or serum/CSF samples for 1 hour at RT, with shaking at 100 rpm on an orbital platform shaker (Unimax 1010, Heidolph, Schwabach, Germany). After washing, incubation with the detection antibody (50 µL/well; 1∶10 biotinylated goat anti-human IgG, F(ab’)_2_; Dianova, Hamburg, Germany; 1∶10.000 in blocking reagent) was performed with 100 rpm shaking at RT for 1 hour. After washing, streptavidin-peroxidase (1∶200 in blocking reagent) was added to the wells and kept in the dark with 100 rpm shaking at RT for 20 min. After a final washing step, the assay was developed using 50 µL/well 3,3′,5,5′-tetramethylbenzidine (TMB; Calbiochem, San Diego, CA, USA) in the dark at RT for 15 min. Thereafter, the reaction was stopped using 20 µL 2N sulfuric acid (Sigma, St. Louis, MO) and absorbance, i.e. the optical density (OD), was measured at 450 nm using an Infinite 200 PRO Microplate Reader (Tecan, Crailsheim, Germany). The OD difference between the triplet mean of α-Syn-coated and uncoated wells of a sample was considered to quantify specific nAbs binding to α-Syn, and was thus used for further analyses.

### Statistical analysis

Normal distribution of data was tested using Kolmogorov-Smirnov-tests. For data showing non-normal distributions non-parametric Whitney-Mann U-tests between groups and Spearman correlations were conducted. For normally-distributed data *t*-tests were calculated. χ^2^-tests were used for dichotomous variables. Fisher transformations and z-tests were used to test differences between Spearman 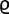 correlation coefficients. The threshold of significance was set to α = 5%.

## Results

### α-Syn-nAbs in PD patients and healthy controls

**Figure 1:**
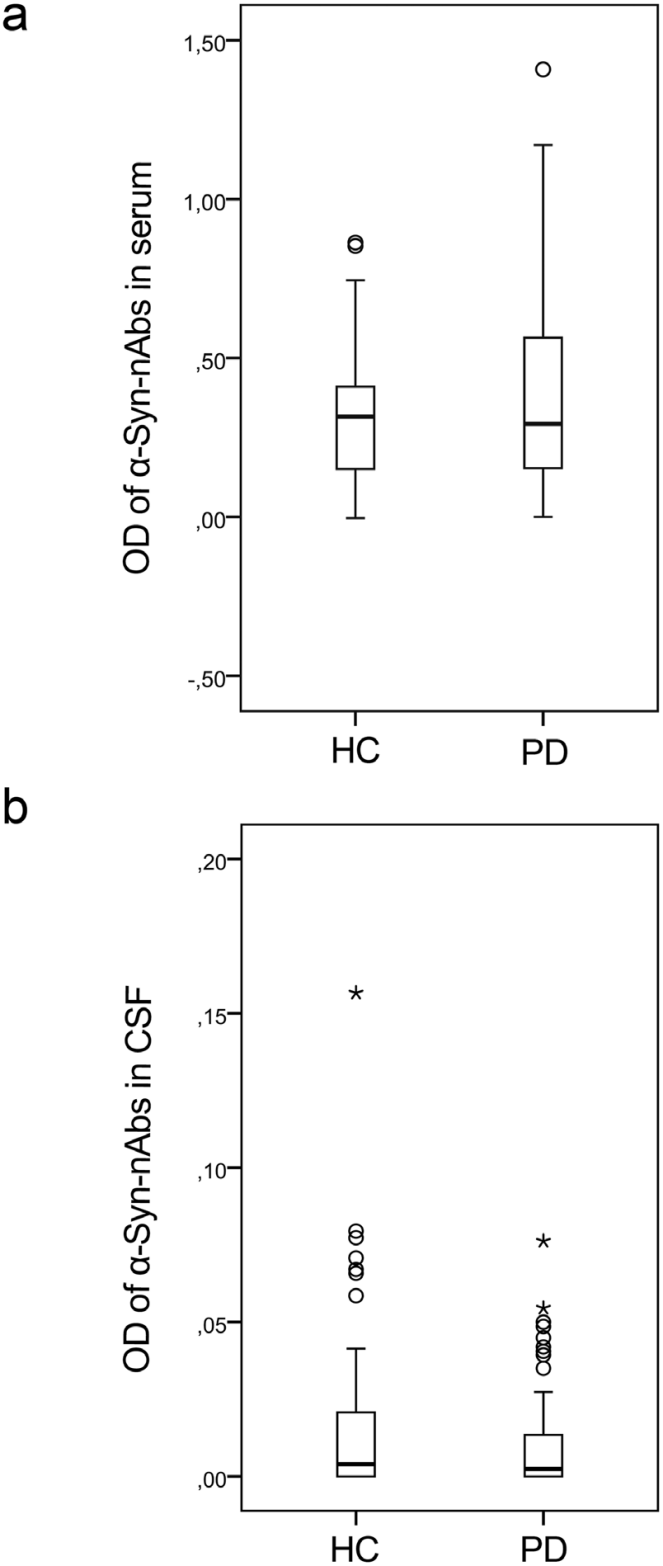
Results of the ELISA of α-Syn-nAbs quantified by optical density (OD) in a) serum and b) cerebrospinal fluid (CSF) of healthy controls (HC) and Parkinson’s disease (PD) patients.

PD patients and HC showed no significant differences in α-Syn-nAbs in serum samples as indicated by OD measurements (U = 2120.5, p = .49, mean ± SD, PD: .38±.30, HC: .31±.21; see figure 1a). PD disease severity as indicated by H&Y was not correlated with α-Syn-nAbs in the PD subgroup (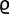 = −.09, p = .49). After excluding patients with H&Y<2 differences in α-Syn-nAbs between HC and PD (n = 52) remained non-significant (U = 1721.0, p = .70).

Similarly, in CSF samples, OD indicating binding of α-Syn-nAbs also did not differ between PD patients and HC (U = 1199.0, p = .29; PD: 0.011±0.018, HC: 0.018±0.031; see figure 1b). In PD patients, OD was not correlated with H&Y (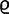 = .17, p = .20), and after excluding patients with H&Y<2 differences to HC remained non-significant (U = 960.5, p = .54).

### Sources of variance in α-Syn-nAbs ELISAs

#### Unspecific background signal

For serum, OD of the α-Syn coated wells was on average 184% higher than the background OD of α-Syn uncoated wells. The background OD (U = 2243.0, p = .88) and signal-background ratios (U = 2192.0, p = .71) did not differ between healthy controls and PD patients.

For CSF, however, the ELISA showed low OD values (mean α-Syn coated OD: 0.085), which were on average 19% higher than the background OD in α-Syn uncoated wells. For 39% of CSF samples the background OD was higher compared to the ODs of the coated wells (without these samples α-Syn coated OD was 37% higher than the background). The number of samples showing a higher OD than background did not differ between healthy individuals and PD patients (χ^2^ = 1.43, p = .23).

#### Free hemoglobin

**Figure 2:**
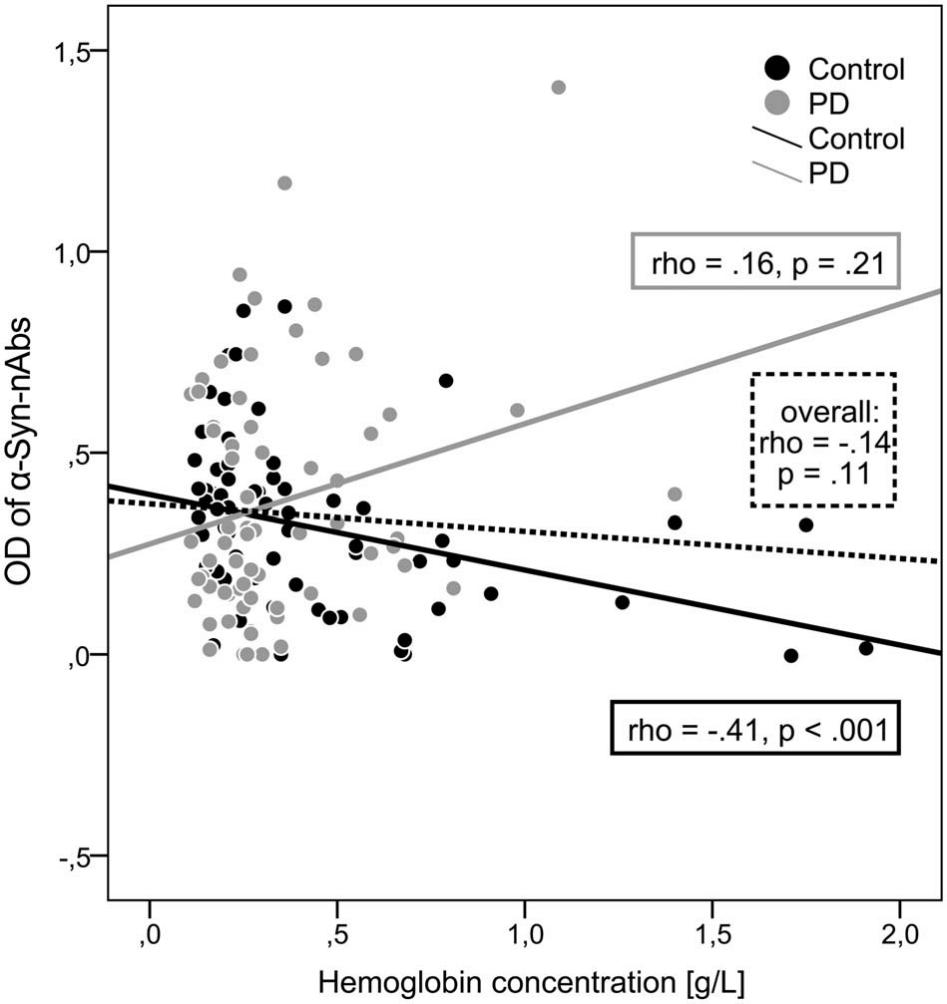
Scatterplot and Spearman correlations (rho, p-values) of α-Syn-nAbs OD and hemoglobin concentrations for the overall sample and separately for healthy controls (black dots/line) and PD patients (grey dots/line).

For serum samples a mean Hb concentration of 0.39±0.33 g/L (range: 0.11–1.91) was measured. The Hb concentrations did not differ between HC and PD patients (U = 2142.0, p = .55). While overall Hb concentrations and OD of α-Syn-nAbs showed no significant correlation (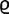 = –.14, p = .11), correlations significantly differed between HC and PD (z = 3.39, p<.001). For HC a significant negative correlation (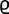 = −.41, p<.001) was observed, whereas no significant correlation was found for the PD sample (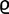 = .16, p = .21) as shown in figure 2.

Within the sensitive detection range of the Hb-concentration measurements only 4 CSF showed non-zero Hb concentrations. Thus, statistical analyses of Hb and CSF OD were omitted.

### Coating and inter-assay variability

**Figure 3:**
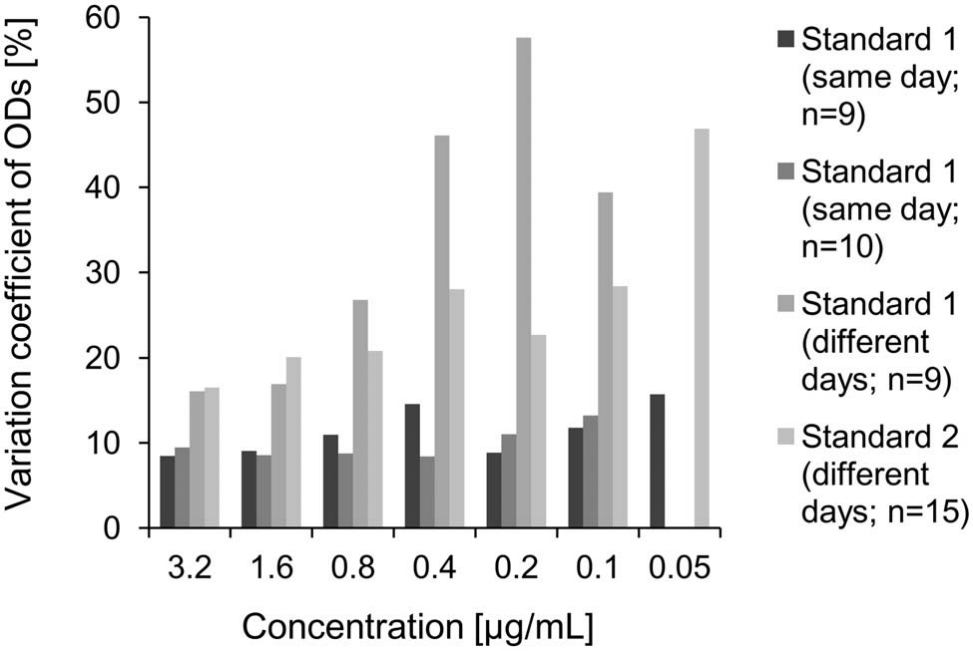
Inter-assay variability of optical densities (ODs) of α-Syn-nAbs standards measured on plates coated with α-Syn on the same day (assays #1, #2) and coated on different days (assays #3, #4).

Comparing the inter-assay variability of ODs of standards assessed using ELISA plates coated with α-Syn on the same versus coating on different days showed substantial differences. Coating on the same day was associated with a reduction of inter-assay variability of at least 50% compared coating on different days. Moreover, for coating on different days the inter-assay variability of ODs increased with increasing dilution of standards, which was not observed for same day coating (figure 3): α-Syn on the same day an inter-assay variability of around 10% (range over different concentrations: 8% to 16%) was shown, whereas for coating on different days a higher inter-assay variability (range over different concentrations: 16% to 58%) was observed, which substantially increased for lower standard concentrations.

For serum control samples, coating on different days showed an inter-assay variability of 17.4% (n = 13; different day coating, OD: 0.7±0.13), whereas same day coating showed a lower inter-assay variability of 8.1% (n = 10, OD: 0.91±0.07) and 15.6% (n = 9, OD: 1.37±0.21).

### α-Syn-nAbs standards

**Figure 4:**
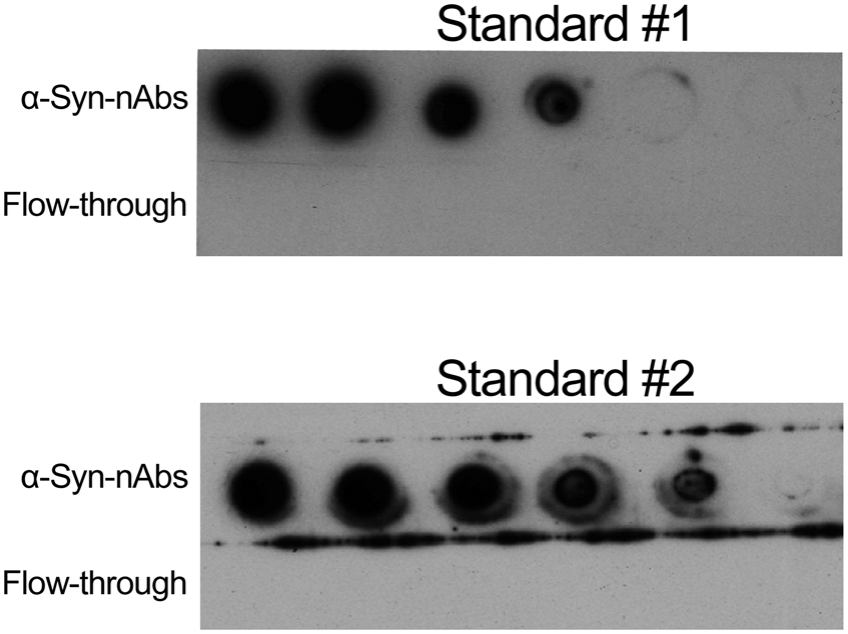
Dot blots of the two α-Syn-nAbs standard preparations of one IvIG batch and the affinity chromatography flow-through showing the specific binding of the purified antibody to recombinant α-Syn in different dilutions.

**Figure 5:**
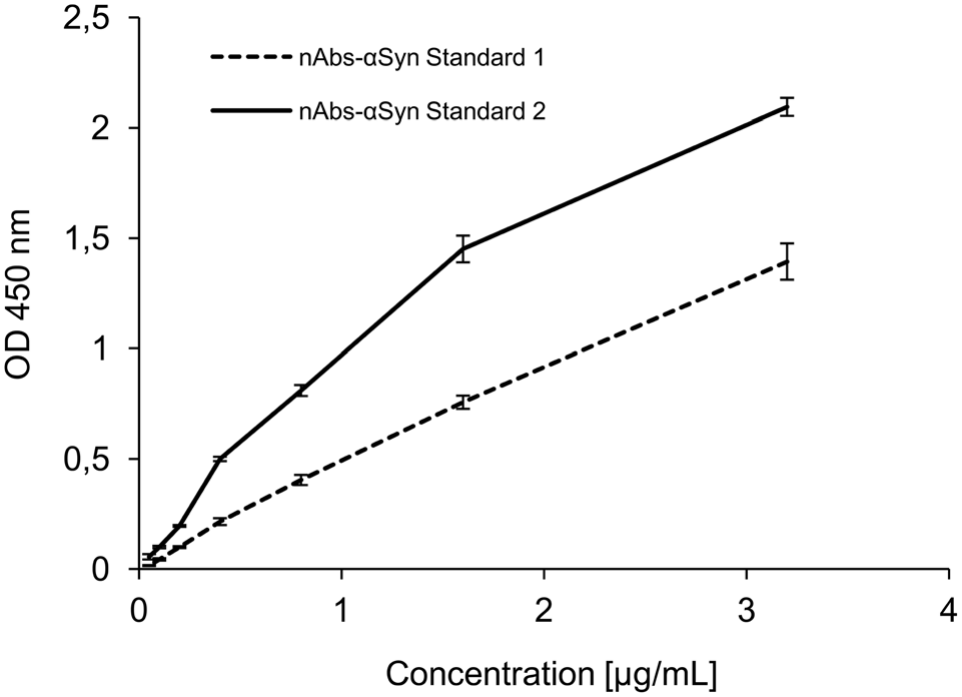
Results of the ELISA of purified α-Syn-nAbs standards from two different preparations of one IvIG batch quantified by optical density (OD; mean ± standard deviation) at 450 **nm.**

Purified standards with concentrations indicated by photo-spectrometric measurements are often used as reference to derive a concentration estimate of a sample of interest in ELISA assays. We investigated purified α-Syn-nAbs from two different purification batches for differences in measured ODs in ELISA assays, i.e. potential standard reference scale differences for sample α-Syn-nAbs concentrations. The two standards were diluted to concentrations of 3.2, 1.6, 0.8, 0.4, 0.2, 0.1 and 0.05 µg/mL and the presence of α-Syn-nAbs in the standards (and absence in the flow-through of affinity chromatography) was confirmed using dot blot (see figure 4). However, using ELISAs OD measurements profoundly differed between standard #1 and #2 as shown in figure 5. Within concentration ranges relevant for the estimation of serum α-Syn-nAbs concentrations, e.g. between 3.2, 1.6 and 0.8 µg/mL, the two standards showed OD differences of 50%, 92% and 100%, respectively.

## Discussion

The present study showed no differences in serum or CSF of naturally occurring alpha-synuclein antibodies (α-Syn-nAbs) between healthy controls (HC) and patients with Parkinson’s disease (PD). Since previous findings of serum α-Syn-nAbs differences between healthy controls and PD patients have been inconsistent, the present study aimed to further investigate potential sources of error variance affecting the quantification of α-Syn-nAbs. We focused on (1) unspecific background of ELISA assays, (2) free hemoglobin in serum samples, (3) α-Syn coating procedures, and (4) purified α-Syn-nAbs standards. In addition to the present findings, we discuss additional potential sources of error variance based on pathological and clinical heterogeneity of PD. Previously, inconsistent findings of increased [32]–[34], decreased [35] and non-significant [14], [29]–[31] differences in serum α-Syn-nAbs of PD patients compared to HC have been reported, and several factors may have to be considered in order to establish α-Syn-nAbs as a valid and reliable PD biomarker.

### Heterogeneity of the clinical sample

PD is a heterogeneous neurodegenerative disease [41] and individuals partly differed in PD subtype as well as disease duration and severity. Thereby, clinically realistic heterogeneity may be present in the PD sample which may however introduce additional (error) variance in α-Syn-nAbs levels of PD patients. Previously, CSF α-Syn, β-Amyloid 1–42, T-tau and P-tau181 levels have been shown to depend on specific PD motor phenotypes [6]. Only PD patients with postural instability-gait disturbance-dominant phenotype but not those with tremor-dominant or equivalent phenotypes showed decreased levels in these markers compared to healthy controls. However, excluding patients with tremor-dominant type (n = 7) did not affect the present results. Current efforts on redefinitions of diagnostic criteria of PD and formal PD subtype definitions [42] may allow for more specific investigations of biomarkers based on pathological processes in PD subtype entities. Moreover, some evidence indicated significant differences in serum α-Syn-nAbs concentrations only in patients with familial but not sporadic forms of PD [30]. Thus, clinical and etiological aspects of PD may need to be considered for PD biomarker analyses.

### Variety of α-Syn and α-Syn-nAbs species

Depending on, for instance, isoform type, phosphorylation and other post-translational modifications, oligomer/aggregation state or environmental factors, such as pH milieu, α-Syn may change its conformation and epitope characteristics [43], which may affect the affinity of α-Syn-nAbs towards α-Syn. In addition, the nAbs, including α-Syn-nAbs are thought to be of oligoclonal origin adding to their epitope variety and avidity [44]. Thereby, a large variety of α-Syn species differing in their physiological and/or pathological role may emerge. Common serum α-Syn-nAbs ELISA assays use recombinant monomeric α-Syn as antigen, and the affinity of quantified α-Syn-nAbs may be restricted to this selected form of α-Syn. Serum α-Syn-nAbs have been shown not to interact with α-Syn oligomers and fibrils or heterogeneous oligomeric α-Syn species, which have each been incubated at different pH values, thereby potentially possessing different conformational properties [34]. While monomer-specific α-Syn-nAbs may indicate one aspect of α-Syn related to PD pathology thus serving as a PD biomarker, other α-Syn species might be additionally informative. For instance, elevated oligomeric α-synuclein CSF levels have been shown in PD patients compared with HC [9]. In addition, recently two distinct α-Syn protofibril strains have been reported, which not only differed in their epitopes and recognition by conformational-specific P-tau antibodies, but also majorly differed in their capacity to cross-seed tau-protein aggregation [45]. Possibly, specific α-Syn-nAbs might recognize these different forms of α-Syn and serve as a PD biomarker or passive immunization agent. ELISA assays might differ in the degree of α-Syn-nAbs binding to (monomeric) α-Syn-coated plates underlying the quantified OD measurement and the free α-Syn-nAbs not contributing to OD measurements indicating α-Syn-nAbs levels. While the complex interactions between α-Syn and α-Syn-nAbs species should be further investigated, they might have contributed to inconsistent findings of (monomer-specific) α-Syn-nAbs differences in serum of PD and HC.

In addition to these potential sources of (error) variance, consideration of other rather methodological aspects (see below) may also vary reproducibility of α-Syn and/or α-Syn-nAbs ELISA findings.

### Unspecific background of ELISA assays

OD values considered unspecific as measured in wells not coated with α-Syn directly impact α-Syn-nAbs quantifications as they are commonly subtracted from OD values in α-Syn coated wells. Therefore, unspecific binding, e.g. peroxidase-conjugated Ig non-specifically binding to polystyrene microtiter plate wells, should be largely blocked by effective agents. Moreover, other potential sources of unspecific OD measurements should be considered including plate reader settings, washing procedure and potential sources of contamination. Importantly, based on the ratio of specific and unspecific OD the error variance may be overly represented in α-Syn-nAbs quantifications.

In the present study, serum samples OD of coated wells was on average 184% higher than the unspecific background. However, CSF samples showed relatively low OD values in α-Syn coated wells, which were only 19% higher than the unspecific background. Thus, unspecific background may, for CSF samples, represent a source of error variance in the previously established and validated serum α-Syn-nAbs ELISA [35] used in the present study. Thus, the findings suggest that this ELISA protocol is not sensitive enough to detect α-Syn-nAbs in CSF samples with a 1∶4 dilution.

### Free hemoglobin levels

Apart from neurons, α-Syn is ubiquitously expressed by many different cell types including erythrocytes, which represent a major source of α-Syn [37], [38]. Disruption of erythrocytes during centrifugation or hemolytic processes may substantially increase monomeric α-Syn in serum or CSF samples [13], [46]. Thereby, α-Syn and α-Syn-nAbs quantifications might be affected in contaminated samples. Previously, blood contamination as indicated by free Hb levels have been shown to significantly increase measured α-Syn in CSF samples [5], [6], [47]. This confounding factor of erythrocytic α-Syn may also partly explain inconsistent findings of serum or plasma α-Syn level differences between PD patients and healthy controls [10]–[18]. For CSF exclusion cut-off thresholds of Hb>0.0002 g/L (200 ng/mL) or >500 erythrocytes per µL CSF before centrifugation have been proposed [6], [11], [47]. These cutoff values may suggest the more than 1000-fold higher serum free Hb levels (with normally around 5 mio. erythrocytes per µL) as a crucial confounding factor potentially affecting α-Syn and α-Syn-nAbs quantification in serum. Possibly, increased erythrocytic α-Syn increase competitive α-Syn-nAbs binding to free α-Syn and plate-bound α-Syn, respectively. Thereby, measured α-Syn-nAbs-related OD would be reduced with increasing free erythrocytic α-Syn and Hb. Interestingly, in the present study a negative correlation between free Hb concentrations and measured α-Syn-nAbs-related OD was observed in the group of healthy subjects (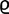 = −.41), whereas in the group of PD patients no significant correlation was present. It can be hypothesized that the diagnostic groups might differ in α-Syn-nAbs affinity towards α-Syn originating from erythrocytes which may contribute to the differential correlations. These findings provide supporting evidence of free Hb as a source of error variance in serum α-Syn-nAbs quantifications, and its differential impact (only decreasing α-Syn-nAbs in healthy controls) might have contributed to the non-significant differences between diagnostic groups.

### α-Syn coating procedures

ELISA inter-assay variability indicates that, while all protocol procedures, chemicals and instruments do not change, measured OD values of the same sample may substantially differ between measurements suggesting additional sources of error variance. Up to 30% inter-assay variability in α-Syn-nAbs-related OD measurement has previously been reported between three independent assays of the same serum sample [36]. Here, α-Syn plate coating procedures affecting the magnitude of α-Syn-nAbs binding might play an important role. Therefore, we investigated the impact of temporal differences in α-Syn coating on the inter-assay variability. Coating α-Syn-nAbs standards on the same day showed acceptable (<20%) inter-assay variability which did not increase with decreasing standard concentrations. However, coating on different days was associated with overly elevated inter-assay variability (>20%), which increased with decreasing standard concentrations. Thus, the present study identified temporal differences in α-Syn coating as an important factor increasing inter-assay variability, and thus error variance, in α-Syn-nAbs assays.

### α-Syn-nAbs standards

ELISA biomarker studies either report OD values or concentrations as inferred by relating the measured OD of samples to those of purified standards, and this lack of consensus may decrease study comparability. The dependence of a sample’s analyte quantification on a reference standard potentially differing between assays and/or studies, however, might be an important caveat of standards. In the present study, we compared the standards from two different purification batches regarding the measured OD. α-Syn binding specificity were confirmed for both standards using dot blots. Here, dot blots or Western blots are important methods to exclude unspecific binding of antibodies. Despite equal concentrations the two standards showed markedly different results in the OD measured in the ELISAs. Within standard concentration ranges crucial for calculating α-Syn-nAbs concentrations from the samples’ OD values the two standards differed by up to 100%. One explanation could be that for each purification batch, a unique constellation of α-Syn-nAbs regarding the overall (monomeric) α-Syn binding affinity is extracted. Thereby, α-Syn-nAbs concentrations derived from standards might be confounded with this additional source of error variance, which may impact study comparability and contribute to inconsistencies between findings. Thus, the present findings suggest the use of OD rather than standard-dependent concentration values in ELISA assays.

### Conclusions

To establish serum or CSF α-Syn-nAbs as a valid and reliable PD biomarker, several potential sources of error variance may have to be considered in addition to the heterogeneity of PD and α-Syn. The present study did not reveal differences in serum or CSF α-Syn-nAbs between PD patients and HC and investigated several potential sources of error variance affecting α-Syn-nAbs quantifications. The following methodological aspects can be concluded from the present findings: (1) α-Syn plate coating may increase inter-assay variability, (2) free serum hemoglobin may (differentially for diagnostic groups) impact serum α-Syn-nAbs levels, and (3) OD measurements may be a more reliable indicator of α-Syn-nAbs levels compared to concentration levels inferred from standards, as α-Syn affinity of α-Syn-nAbs standards may depend upon purification. Future α-Syn-nAbs and α-Syn biomarker studies should consider these methodological aspects.
